# Guidelines for Neuroprognostication in Adults with Guillain–Barré Syndrome

**DOI:** 10.1007/s12028-023-01707-3

**Published:** 2023-03-25

**Authors:** Katharina M. Busl, Herbert Fried, Susanne Muehlschlegel, Katja E. Wartenberg, Venkatakrishna Rajajee, Sheila A. Alexander, Claire J. Creutzfeldt, Gabriel V. Fontaine, Sara E. Hocker, David Y. Hwang, Keri S. Kim, Dominik Madzar, Dea Mahanes, Shraddha Mainali, Juergen Meixensberger, Oliver W. Sakowitz, Panayiotis N. Varelas, Thomas Westermaier, Christian Weimar

**Affiliations:** 1 Departments of Neurology and Neurosurgery, College of Medicine, University of Florida, Gainesville, FL USA; 2 Department of Neurosurgery, Denver Health Medical Center, Denver, CO USA; 3 Departments of Neurology, Anesthesiology, and Surgery, University of Massachusetts Chan Medical School, Worcester, MA USA; 4 Department of Neurology, University of Leipzig, Leipzig, Germany; 5 Departments of Neurology and Neurosurgery, University of Michigan, Ann Arbor, MI USA; 6 School of Nursing, University of Pittsburgh, Pittsburgh, PA USA; 7 Department of Neurology, University of Washington, Seattle, WA USA; 8 Departments of Pharmacy and Neurosciences, Intermountain Health, Salt Lake City, UT USA; 9 Department of Neurology, Mayo Clinic, Rochester, MN USA; 10 Department of Neurology, University of North Carolina, Chapel Hill, NC USA; 11 Department of Pharmacy Practice, University of Illinois, Chicago, IL USA; 12 Department of Neurology, University of Erlangen-Nuremberg, Erlangen, Germany; 13 Departments of Neurology and Neurosurgery, University of Virginia Health, Charlottesville, VA USA; 14 Department of Neurology, Virginia Commonwealth University, Richmond, VA USA; 15 Department of Neurosurgery, University of Leipzig, Leipzig, Germany; 16 Department of Neurosurgery, Neurosurgery Center Ludwigsburg-Heilbronn, Ludwigsburg, Germany; 17 Department of Neurology, Albany Medical College, Albany, NY USA; 18 Department of Neurosurgery, University of Würzburg, Würzburg, Germany; 19 Institute of Medical Informatics, Biometry, and Epidemiology, University Hospital Essen, Essen and BDH-Clinic Elzach, Essen, Germany; 20 BDH-Clinic Elzach, Elzach, Germany

**Keywords:** Guillain–Barré syndrome, Polyradiculoneuropathy, Prognosis, Outcome

## Abstract

**Background:**

Guillain–Barré syndrome (GBS) often carries a favorable prognosis. Of adult patients with GBS, 10–30% require mechanical ventilation during the acute phase of the disease. After the acute phase, the focus shifts to restoration of motor strength, ambulation, and neurological function, with variable speed and degree of recovery. The objective of these guidelines is to provide recommendations on the reliability of select clinical predictors that serve as the basis of neuroprognostication and provide guidance to clinicians counseling adult patients with GBS and/or their surrogates.

**Methods:**

A narrative systematic review was completed using Grading of Recommendations Assessment, Development and Evaluation (GRADE) methodology. Candidate predictors, including clinical variables and prediction models, were selected based on clinical relevance and presence of appropriate body of evidence. The Population/Intervention/Comparator/Outcome/Time frame/Setting (PICOTS) question was framed as follows: “When counseling patients or surrogates of critically ill patients with Guillain–Barré syndrome, should [predictor, with time of assessment if appropriate] be considered a reliable predictor of [outcome, with time frame of assessment]?” Additional full-text screening criteria were used to exclude small and lower quality studies. Following construction of an evidence profile and summary of findings, recommendations were based on four GRADE criteria: quality of evidence, balance of desirable and undesirable consequences, values and preferences, and resource use. In addition, good practice recommendations addressed essential principles of neuroprognostication that could not be framed in PICOTS format.

**Results:**

Eight candidate clinical variables and six prediction models were selected. A total of 45 articles met our eligibility criteria to guide recommendations. We recommend bulbar weakness (the degree of motor weakness at disease nadir) and the Erasmus GBS Respiratory Insufficiency Score as moderately reliable for prediction of the need for mechanical ventilation. The Erasmus GBS Outcome Score (EGOS) and modified EGOS were identified as moderately reliable predictors of independent ambulation at 3 months and beyond. Good practice recommendations include consideration of both acute and recovery phases of the disease during prognostication, discussion of the possible need for mechanical ventilation and enteral nutrition during counseling, and consideration of the complete clinical condition as opposed to a single variable during prognostication.

**Conclusions:**

These guidelines provide recommendations on the reliability of predictors of the need for mechanical ventilation, poor functional outcome, and independent ambulation following GBS in the context of counseling patients and/or surrogates and suggest broad principles of neuroprognostication. Few predictors were considered moderately reliable based on the available body of evidence, and higher quality data are needed.

**Supplementary Information:**

The online version contains supplementary material available at 10.1007/s12028-023-01707-3.

## Introduction

Guillain–Barré syndrome (GBS) is an acute immune-mediated polyradiculoneuropathy that can affect all myelinated nerves [1]. GBS is the most common cause of acute flaccid paralysis, with an estimated one to two cases per 100,000 person-years [2]. Incidence is higher in men and rises with age [3–6]. Patients with GBS typically present with sensory symptoms and progressive limb weakness extending to arms and cranial nerves. The acute period is often characterized by rapid progression of symptoms over days, reaching their maximum within 2–4 weeks [7]. However, the clinical presentation and course of GBS is highly variable, and distinct clinical variants exist [8]. The diagnosis of GBS is mainly based on history and clinical features and is often supported by electrophysiological studies and cerebrospinal fluid examination [9–11]. Electrophysiological studies provide evidence of peripheral nervous dysfunction and distinguish between demyelinating, motor axonal, and sensory axonal polyneuropathies [12]. Intravenous immunoglobulin and plasmapheresis have proven to be effective treatments for GBS [13].

Mortality rates in GBS range between 1 and 13% [14, 15]. The most common causes of death in patients with GBS are respiratory and cardiovascular complications, and for patients requiring mechanical ventilation, mortality rates up to 20% have been observed [16, 17]. On the other hand, for the majority, GBS is a reversible disease with usually favorable prognosis. During the acute phase, approximately 10–30% of all patients with GBS require mechanical ventilation [18]. Diaphragmatic weakness is the main reason for respiratory failure in neuromuscular patients, but respiratory failure may also be the result of pulmonary complications, such as aspiration and pneumonia, which may occur because of oropharyngeal muscle weakness or poor cough strength [19]. Various predictors for respiratory failure have been described. The presence of several predictors increases the risk for needing mechanical ventilation [7], an observation that led to the development of prediction scores. After the acute phase, focus usually shifts to restoration of motor strength and function. Prognostication during the hospital course is essential; however, guidance is scarce. The objective of these guidelines is to provide recommendations on the reliability of select clinical predictors that serve as the basis of neuroprognostication and provide guidance to clinicians counseling adult patients with GBS or their surrogates.

### Scope, Purpose, and Target Audience

The scope of these Grading of Recommendations Assessment, Development and Evaluation (GRADE) guidelines is the prognostication of neurological outcome in adult patients with GBS. The purpose of these guidelines is to provide evidence-based recommendations on the reliability of predictors of neurological outcome in adult patients with GBS to aid clinicians in formulating a prognosis. The target audience consists of clinicians responsible for such counseling.

### How to Use These Guidelines

These guidelines provide recommendations on the reliability of select demographic and clinical variables, as well as prediction models, when counseling families and surrogates of critically ill patients with GBS. We categorized these predictors as reliable, moderately reliable, or not reliable. We based this categorization on a GRADE-based assessment of certainty in the body of evidence, as well as effect size (quantification of predictor accuracy) across published studies, as detailed in Supplementary Appendix 1 and Table 1. Reliable predictors and prediction models for the purposes of these guidelines may be used to formulate a prognosis when the appropriate clinical context is present in the absence of potential confounders. These are predictors with clear, actionable thresholds or clinical/radiographic definitions and a low rate of error in prediction of outcomes, with at least moderate certainty in the body of evidence. When prognosis is formulated on the basis of one or more reliable predictors, the clinician may describe the outcome as “very likely” during counseling. Given the inherent limitations in neuroprognostication research, the clinician must nevertheless acknowledge the presence of uncertainty—even if low—in the prognosis during counseling. Moderately reliable individual predictors may be used for prognostication only when additional reliable or moderately reliable individual predictors are present, in addition to the appropriate clinical context. These are also predictors with clear, actionable thresholds or clinical/radiographic definitions and a low rate of error in prediction of outcomes, but with lower certainty in the body of evidence, frequently as a result of smaller studies that result in imprecision or other risk of bias, often rooted in methodology. When the prognosis is formulated on the basis of multiple moderately reliable predictors, the clinician may describe the outcome as “likely” during counseling but must acknowledge “substantial” uncertainty in the prognosis. Moderately reliable clinical prediction models that generate predicted probabilities of outcomes, in contrast, may be used for prognostication during counseling of patients with GBS and their surrogates in the absence of other reliable or moderately reliable predictors. However, it is recommended that the clinician describes the predicted probability of the outcome as “an objective estimate only, subject to considerable uncertainty.” Although the panelists recognize that those predictors that do not meet the criteria to be described as reliable or moderately reliable are often used by clinicians in formulating their subjective impressions of prognosis, they have nevertheless been deemed not reliable for the purposes of these guidelines and cannot be formally recommended for prognostication on their own. Variables deemed not reliable, however, may be a component of reliable or moderately reliable prediction models.

**Table 1:** Predictor characterization and use: reliable and moderately reliable predictors

Category of predictor/model	GRADE criteria					Point estimates of accuracy in the body of evidence	Use during counseling of patients or surrogates?	Presence of additional specific reliable or moderately predictors required for use during counseling	Suggested language during counseling of patients or surrogates	
	Risk of bias	Inconsistency	Imprecision	Indirectness	Quality of evidence, overall				Likelihood of outcome	Disclaimer of Uncertainty during counseling
Reliable	One downgrade permitted	Downgrade not permitted	Downgrade not permitted	Downgrade not permitted	Moderate or High	High. Prediction models require AUC > 0.8, no evidence of miscalibration in external validation studies	Yes	Preferred, but not absolutely required	“Very likely” or for clinical prediction models use predicted probability of outcome	Present, but low
Moderately reliable individual predictors	One downgrade permitted	Downgrade not permitted	One downgrade permitted	One downgrade permitted	Any	High	Yes	Yes	“Likely”	Substantial
Moderately reliable clinical prediction models	One downgrade permitted	Downgrade not permitted	One downgrade permitted	One downgrade permitted	Any	High. Prediction models require AUC > 0.7, some miscalibration allowed in external validation studies	Yes	No	Use predicted probability of outcome	“The predicted probability is an objective estimate, subject to considerable uncertainty”
Not reliable	Downgrade permitted	Downgrade permitted	Downgrade permitted	Downgrade permitted	Any	Any	No^a^	Not applicable	Not applicable	Not applicable

^a^Many predictors designated “not reliable” are practically used by clinicians in formulating and communicating real-world subjective impressions of prognosis. The purpose of these guidelines is to identify predictors, if any, that meet reliable or moderately reliable criteria

*AUC* Area under the curve

## Methods

The Quality in Prognostic Studies (QUIPS) RoB instrument was used to evaluate studies of individual prognostic variables, and the Prediction model Risk Of Bias ASsessment Tool (PROBAST) instrument used to evaluate studies of clinical prediction models. An in-depth description of the methodology used in these guidelines can be found in the appendix (Supplementary Appendix 1, Methodology).

### Selection of Guideline Questions

Candidate predictors were selected based on clinical relevance and the presence of an appropriate body of literature. Candidate predictors and prediction models were considered “clinically relevant” if in the opinion of the content experts and guideline chairs, the predictor or components of the prediction models were (1) accessible to clinicians, although universal availability was not required, and (2) likely to be considered by clinicians when formulating a neurological prognosis for critically ill patients with GBS. An appropriate body of literature was considered present if a clinical variable fulfilled two criteria: (1) evaluated in at least two published studies that included a minimum of 35 study participants and (2) established as an independent predictor in a multivariate analysis. An appropriate body of literature was considered present for clinical prediction models with at least one external validation study of at least 50 patients in addition to the initial report on development of the model (also with a minimum of 50 patients).

Based on these criteria, the following candidate predictors were selected:

Clinical variables:

1. Age
2. Presence of bulbar weakness
3. Degree of motor weakness
4. Progression of motor weakness
5. Neck weakness
6. Dysautonomia
7. Need for mechanical ventilation
8. Electrophysiologic subtype

Clinical prediction models:

1. Erasmus GBS Respiratory Insufficiency Score (EGRIS) score (mechanical ventilation)
2. Neck weakness, single breath count, and bulbar palsy score (mechanical ventilation)
3. Sharshar model (mechanical ventilation)
4. Ning Score (mechanical ventilation nomogram [MVN])
5. Erasmus GBS Outcome Score (EGOS) (independent ambulation)
6. Modified EGOS (mEGOS) (independent ambulation)

The Population/Intervention/Comparator/Outcome/Time frame/Setting (PICOTS) question was then framed for the specific candidate predictors as follows: “When counseling patients or surrogates of critically ill patients with Guillain–Barré syndrome, should [predictor, with time of assessment if appropriate] be considered a reliable predictor of [outcome, with time frame of assessment]?”.

### Selection of Outcomes

Neuroprognostication in the context of GBS is focused on the likelihood of respiratory failure and protracted course of illness, as well as the prediction of recovery of function. Outcomes were rated on a numeric scale from 1 to 9, indicating degree of importance from low to high, by the two primary content experts and the public representative. The outcomes considered “critical” for the systematic review and subsequent formulation of recommendations were the following: (1) need for mechanical ventilation (average rating 8.00) assessed within 14 days after disease onset, (2) independent ambulation (average rating 8.33) assessed at or beyond 3 months from admission, and (3) functional outcome (average rating 8.67) assessed at or beyond 3 months from admission. Prognostication of mortality plays less of a role in GBS, as opposed to many other neurocritical illnesses, as the overall mortality is relatively low. Although prolonged mechanical ventilation was considered and deemed relevant by the panel, the lack of sufficient data did not allow for its inclusion as selected outcome.

Functional outcome assessment is highly variable between studies, with different time points and assessment methods as well as lack of standardized outcome measures [20]. Functional outcome is most commonly assessed using the GBS disability scale (GBSDS), also known as the Hughes scale [21] (see Table 2). This scale is focused on motor function, and in many studies, outcomes are dichotomized between good (GBSDS 0–2) and poor (GBSDS 3–6). Other acceptable measures of functional outcome in our systematic review included the modified Rankin scale and any other equivalent assessment tool. Other commonly used outcome end points include the ability to walk independently. More recently, the focus has shifted to assessment of longitudinal assessment of quality of life [22] and functional assessment scales [23], but data using these instruments are not yet available. Follow-up in most studies is limited to 6 months or 1 year, which may result in an overestimation of poor outcomes, as longer times are often needed to regain more function, and long-term recovery from severe GBS is possible [24]. On the other hand, many of the data for outcome assessment stem from clinical trial cohorts, which may result in an overestimate of good recovery. Additionally, intensive rehabilitation may improve outcome [25, 26], but the role of rehabilitation in most outcomes studies has not been evaluated. Similarly, impact of factors such as nutrition may play a role that is yet to be elucidated [27].

**Table 2:** Guillain–Barré syndrome disability scale

0	Healthy
1	Minor symptoms or signs of neuropathy but capable of manual work
2	Able to walk without support of a stick
3	Able to walk with a stick, appliance of support
4	Confined to bed or chair bound
5	Requiring assisted ventilation
6	Death

Adapted from Hughes et al. [21]

### Systematic Review Methodology

**Fig. 1:**
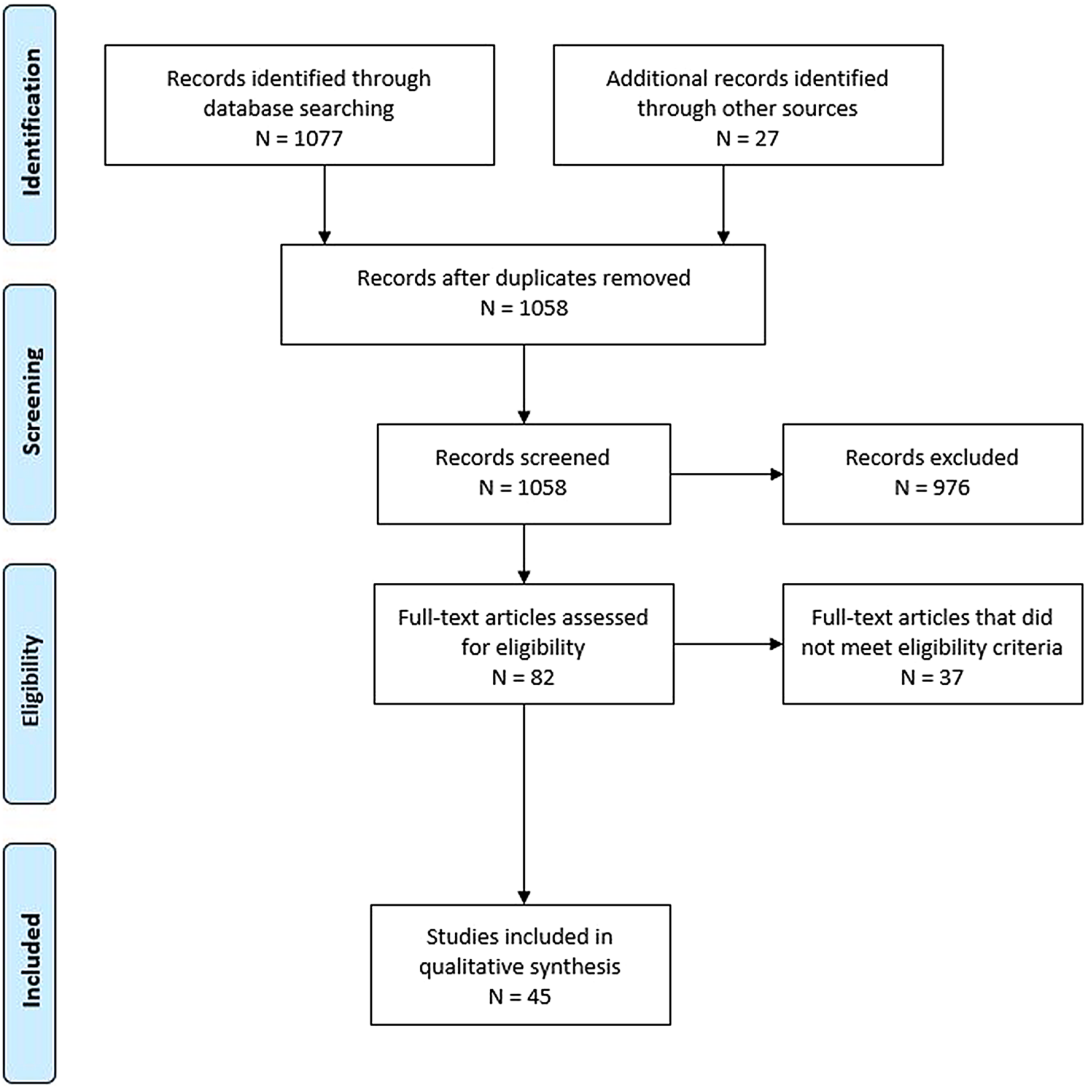
Preferred Reporting Items for Systematic Reviews and Meta-Analyses (PRISM) flow diagram for study screening and selection

An in-depth description of systematic review methodology for these guidelines can be found in the Supplemental Material. Databases searched included Medline via PubMed, Embase, Web of Science, and the Cochrane Database of Systematic Reviews. The in-depth literature search first conducted on February 20, 2019, included literature from 1946 onwards and was last updated on 3/16/2022. The librarian search string used for this systematic review is in Supplementary Appendix 2. Full-text screening was performed with the following exclusion criteria: studies with sample sizes less than 35 (to broaden the review in light of the overall limited body of data on single predictors including more than 50 study participants), studies focused only on a highly selected subgroup (such as ventilated patients or chronic polyneuropathies), studies of predictors not evaluated in multivariate analysis, studies focused on a genetic polymorphism as a predictor, and studies of clinical prediction models that did not report model discrimination. Studies of laboratory biomarkers were included only if the biomarker was considered clinically relevant and had been evaluated in more than one published study that met all other criteria. Studies were therefore screened for several sources of bias while selecting full-text articles for further review. The Preferred Reporting Items for Systematic Reviews and Meta-Analyses flow diagram for study screening and selection is shown in Fig. 1. We included a total of 45 studies in our data synthesis.

A summary of individual studies of predictors and models is presented in the Supplementary Appendix 3. The GRADE evidence profile and summary of findings table for predictors of mechanical ventilation, functional outcome, and independent ambulation is presented in Table 3.

**Table 3:** GRADE Evidence profile/summary of findings table for individual predictors and models of mechanical ventilation, functional outcome, and independent ambulation

Outcome	Predictor	Quality of Evidence					Summary of findings (narrative of effect size)
		RoB	Inconsistency	Indirectness	Imprecision	QoE summary	
Mechanical ventilation	Bulbar weakness	↓		↓	↓	Very low	OR ranging from 1.96 to 48.08 (of note, large 95% CI); in some studies, facial-bulbar not separated/cranial nerve dysfunction and bulbar weakness used interchangeably
Mechanical ventilation	Degree of motor weakness (limb strength less than antigravity, MRC grade) on admission or nadir	↓			↓	Low	OR ranging from 2.5 to 87 (of note, large 95% CI) depending on threshold for MRC sum score
Mechanical ventilation	Rapid disease progression	↓		↓	↓	Very low	OR estimates not consistently performed; ranging from 1.68 to 25; different definition of rapid progression
Mechanical ventilation	Neck weakness	↓	↓	↓	↓	Very low	OR 4.34 to 9.86 (large 95% CI)
Mechanical ventilation	Autonomic dysfunction	↓↓	↓		↓	Very low	Variable definition of autonomic dysfunction
Mechanical ventilation	EGRIS	↓				Moderate	AUC 0.82; in another validation cohort, AUC was 0.86 (0.80–0.93). Lowest AUC reported 0.63
Mechanical ventilation	Sharshar Score	↓			↓	Low	AUC 0.81 (0.72–0.90) Only one validation study
Mechanical ventilation	New normogram	↓			↓	Low	AUC 0,85, C-index 0.86 (95% CI 0.77–0.94) Only one validation study
Functional outcome	Need for mechanical ventilation	↓	↓			Low	OR 2.5–16 (for poor outcome)
Functional outcome	Axonal electrophysiologic subtype	↓	↓		↓	Very low	OR 2–14.45
Functional outcome	Age	↓			↓	Low	OR 1.3–10.3 (dependent on age categories or per year)
Functional Outcome	Disability grade at nadir	↓			↓	Low	OR 2.9–8.88, variable scales used to assess both intake and outcome
Independent ambulation	Disability grade at nadir (MRC sum score, GBSD score)	↓			↓	Low	OR to 3–4 for MRC sum score > 40
Independent ambulation	Age	↓			↓	Low	OR 1.08–3.8 (dependent on age categories or per year)
Independent ambulation	Progression of weakness (speed)	↓		↓	↓	Very low	OR 3.8–8.6 (for poor outcome)
Independent ambulation	EGOS	↓				Moderate	AUC 0·85, 95% CI 0·81–0·89
Independent ambulation	mEGOS	↓				Moderate	after 1 week AUC 0.84–0.87 and at admission the AUC is 0.73–0.77. In another validation cohort, the AUC was 0.75 (0.70–0.80) after 1 week and 0.74 (0.69–0.79) at admission

*OR* Odds ratio; *CI* Confidence interval; *MRC* Medical research council; *EGRIS* Erasmus Guillain Barré Syndrome Respiratory Insufficiency score; *AUC* Area under the curve; *EGOS* Erasmus Guillain Barré Syndrome Outcome Score; *mEGOS* modified Erasmus Guillain Barré Syndrome Outcome Score

### Evidence to Recommendation Criteria

#### Quality of Evidence/Certainty in the Evidence and Effect Size

For the purpose of these guidelines, predictors described as “reliable” have both a higher overall certainty in the evidence and a greater effect size than “moderately reliable” predictors (Table 1). For “reliable” predictors and prediction models, one downgrade was permitted for risk of bias but none for inconsistency, imprecision, or indirectness, and the overall quality of evidence had to be high or moderate. “Reliable” prediction models were required to demonstrate an area under the curve (AUC) of > 0.8 and no evidence of miscalibration in external validation studies that reported calibration. Single downgrades within each of the domains of risk of bias, imprecision, and indirectness were permitted for “moderately reliable” predictors, but a downgrade for inconsistency was not. In addition, “moderately reliable” prediction models were required to demonstrate an AUC > 0.7, and some miscalibration in some external populations was allowed given the lower risk of withdrawal of life support in this disease. Predictors that did not fit “reliable” or “moderately reliable” criteria were classified as “not reliable.”

#### Balance of Desirable and Undesirable Consequences

A desirable consequence of accurate prediction of the need for mechanical ventilation or protracted illness and disability is the ability of patients and/or surrogates and the clinical team to plan appropriate consecutive care including rehabilitation measures. Accurate prognostication also allows patient-centered goal setting. The disadvantages of an inaccurate prediction of a poor outcome (i.e., a false positive prediction of poor outcome) include fear and anxiety about the future, limitations of care, or even withdrawal of life support in an individual who could otherwise have made a meaningful recovery. The expert panel’s consensus considered withdrawal of life support measures based on unreliable predictors in patients with GBS less likely compared with that in patients with other neurointensive conditions, such as severe traumatic brain injury, stroke, or cardiac arrest.

#### Values and Preferences

The panel agreed that most individuals, as well as their families and surrogates, would likely prefer an inaccurate prediction of the need for mechanical ventilation over unawareness of such possibility. The patient representative observed that patients with GBS are often conscious and able to participate in bedside discussions with health care providers. The patient representative emphasized the value of being informed and planning for further necessary care, which may include intubation, a prolonged hospital and rehabilitation stay, and caregiver support for activities of daily living. The patient representative also described the psychological impact of an unexpected clinical decline, compared to anticipation of potential deterioration, and counseling by health care providers, which permits emotional adjustment to the likelihood of a more difficult clinical course.

#### Resource Use

Most diagnostic elements required for the selected predictors and prediction models are within the standard of care for the management of GBS and thereby do not require additional expenditure of resources. Additionally, an accurate prediction of outcome may allow for more informed and efficient resource use, such as early tracheostomy to facilitate an earlier transition in care and reduction in intensive care unit length of stay. The use of resources was therefore thought to favor consideration of a predictor or prediction model during prognostication, contingent on confidence in its predictive accuracy.

### Good Practice Statements

These statements reflect good clinical practice that in the judgment of the content experts lacked a meaningful body of direct evidence to answer a question framed in PICOTS format, often because of insufficient clinical equipoise. Explicit statement of these clinical practice principles was nevertheless considered essential to provide context as well as appropriate guidance.

1. Good practice statement 1: We recommend that assessment of the neurological prognosis for patients with GBS should simultaneously focus on two phases of the illness: the acute course of hospitalization and long-term recovery (strong recommendation, evidence cannot be graded).
  Rationale: The course of GBS in patients requiring admission to a critical care unit is framed by three time points: diagnosis, discharge, and recovery [28]. The acute phase is characterized by possible compromise of respiratory function, autonomic dysfunction, and cardiovascular complications [29]. The postacute phase will focus on recovery of motor and other functions but often differs for patients who require mechanical ventilation during the acute phase [30]. Providing outlook on all phases is supported by analysis of patients’ perception [28].
2. Good practice statement 2: Guillain–Barré syndrome is a dynamic illness during the acute course, and rapid progression may occur. We recommend that neuroprognostication in patients with GBS in the acute phase should include a discussion about the possible need for mechanical ventilation and enteral nutrition bypassing the oral route (strong recommendation, evidence cannot be graded).
  Rationale: Ten to thirty percent of all patients with GBS will require mechanical ventilation during the disease course [18]. Once mechanically ventilated, seven of ten patients will require prolonged mechanical ventilation through a tracheostomy [31]. This will usually also require percutaneous gastrostomy for nutrition. Requirement of ventilatory support directly affects planning of rehabilitation and has been associated with longer rehabilitation needs [30].
3. Good practice statement 3: We recommend that prognostication for patients with GBS should be performed with consideration of the complete clinical condition and never based on a single variable (strong recommendation, evidence cannot be graded).
  Rationale: As outlined above, there are a number of limitations to the available body of evidence in factors of prognostication in GBS. Although prediction models have been developed to take into account several factors [32], other considerations, including complications during hospitalization [29, 33, 34] and factors that may affect quality of life subsequently [22, 35], are important to address and consider.

#### Recommendations: Clinical Variables as Predictors

Although several combinations of predictors and outcomes were identified as clinically relevant, in the section below, we only present recommendations for the predictor/outcome combinations with a sufficient body of evidence that met our criteria, described in the Systematic review methodology section.

#### Outcome: Need for Mechanical Ventilation Within 14 days of Disease Onset

1. Question 1: When counseling patients or surrogates of critically ill patients with GBS, should bulbar weakness assessed on presentation or during the early course of hospital admission be considered a reliable predictor of the need for mechanical ventilation within 14 days of disease onset?
  *Description of the predictor*: Bulbar weakness refers to bilateral impairment of function of the lower cranial nerves (i.e., IX, X, XI, and XII). In the setting of GBS, such weakness occurs because of polyneuropathy affecting these lower cranial nerves outside the brainstem. Examination of bulbar nerve function as part of routine neurological assessment in patients with GBS is conducted on admission and subsequently throughout hospitalization. When assessing bulbar weakness as a predictor of the need for mechanical ventilation, its function is routinely assessed on admission or presentation, as well as early (i.e., the first few days) during the hospital course. Overall, oropharyngeal weakness occurs in about half of patients with GBS [36].
  *Recommendation*: When counseling patients and/or family members/surrogates of patients with acute GBS, we suggest that bulbar weakness be considered a moderately reliable predictor of the need for mechanical ventilation within 14 days of disease onset (weak recommendation; very low-quality evidence).
  *Rationale*: The body of evidence was downgraded for risk of bias, with various studies demonstrating potential bias in the QUIPS domains of study attrition, prognostic factor measurement, outcome measurement, and statistical analysis and reporting, rendering most studies with overall moderate risk of bias. The body of evidence was further limited by indirectness and imprecision but had overall consistency of the association of bulbar weakness with the need for mechanical ventilation [37–47]. Imprecision was due to large confidence intervals, and indirectness was due to the variable definition of “bulbar weakness” with assessment of either individual, some, or all cranial nerves in several of the included studies. Although the false positive rate may be considerable, discussing the possible need for mechanical ventilation early during hospitalization will allow for adjustment of expectations and hence favors considering the predictor for prognostication while counseling patients, families, and surrogates.
2. Question 2: When counseling patients or surrogates of critically ill patients with GBS, should the degree of motor weakness be considered a reliable predictor of the need for mechanical ventilation within 14 days of disease onset?
  *Description of the predictor*: The degree of motor weakness can range from very mild paraparesis or tetraparesis to tetraplegia. Most patients progress to some degree of weakness in both arms and legs [36]. The degree of motor weakness is most commonly assessed using the Medical Research Council (MRC) grading scale or the MRC sum score, which was developed for patients with GBS and has good interrater reliability [48].
  *Recommendation*: When counseling patients and/or family members/surrogates of patients with acute GBS, we suggest that the degree of motor weakness (as assessed on admission to the hospital or during the acute phase at disease nadir by the MRC grade) indicating less than antigravity strength in arms and legs be considered a moderately reliable predictor of the need for mechanical ventilation within 14 days of disease onset (weak recommendation; low-quality evidence).
  *Rationale*: The body of evidence was downgraded for risk of bias, with various studies demonstrating potential bias in the QUIPS domains of study attrition, prognostic factor measurement, outcome measurement, and statistical analysis and reporting, rendering most studies with overall moderate risk of bias. The body of evidence was further limited by imprecision. One study did not find an association of the degree of motor weakness with the need for mechanical ventilation; however, in this study, only 14% received standard-of-care treatment for GBS [49]. An additional important consideration for use of the degree of motor weakness as a predictor is that different studies assessed the degree of motor weakness variably, both in point of time—including on admission [38, 44, 47] or during the acute phase at disease nadir [43, 50, 51] or not further specified [45, 52]—and with variable assessment tools, either by the MRC sum score or the MRC grading scale (score of 1–5) [44, 52]. Furthermore, the degree of weakness was categorized differently, with various groups of MRC sum score strength or lack of antigravity strength of various muscle groups. For example, in some studies, the MRC sum scores were categorized as 0–20, 21–40, and 41–60 [45], and there were different average MRC scores for admission (around 30) versus during nadir (MRC sum score < 20) [53]. Similarly, limb strength assessment may focus on both upper and lower extremity limbs or select major muscle groups [47, 54]. Different evaluations all agreed on association of higher degree of weakness with risk of respiratory failure. However, there is variability in data regarding the threshold definition for degree of weakness. Nevertheless, discussing the possible need for mechanical ventilation early during hospitalization in relation to observation of the degree of motor weakness will allow for adjustment of expectations and hence favors considering the predictor for prognostication while counseling patients, families, and surrogates.
3. Question 3: When counseling patients or surrogates of critically ill patients with GBS, should rapid progression of disease be considered a reliable predictor of the need for mechanical ventilation within 14 days of disease onset?
  *Description of the predictor*: The rate of disease progression is quantified differently in different studies, focusing on the duration of time for a patient to reach either the disease nadir, the need for admission to the hospital, or a prespecified functional disability [55].
  *Recommendation*: When counseling patients and/or family members/surrogates of patients with acute GBS, we suggest that rapid progression of disease (i.e., time from symptom onset to nadir or significant progression of motor weakness over the first few days of presentation) alone, assessed on presentation or during the early course of hospital admission, not be considered a reliable predictor of the need for mechanical ventilation within 14 days of disease onset (weak recommendation; very low-quality evidence).
  *Rationale*: The body of evidence was downgraded for risk of bias, with various studies demonstrating potential bias in the QUIPS domains of study attrition, prognostic factor measurement, outcome measurement, and statistical analysis and reporting, rendering most studies with overall at least moderate risk of bias. The body of evidence was further limited by imprecision and indirectness. Imprecision was due to large confidence intervals with inconsistent determination of risk estimates, and indirectness was based on estimates for speed of progression stemming from the time interval between ability to walk and bedbound state and the time from symptom onset to presentation or admission. Five studies assessing the duration of progression found an association with increased risk for the need of mechanical ventilation [38, 39, 43, 44, 56], with varying definitions of “rapid progression” and lack of adjustment for the time from onset of symptoms to admission, rendering this factor too vague to serve as a reliable factor of prognostication.
4. Question 4: When counseling patients or surrogates of critically ill patients with GBS, should neck weakness be considered a reliable predictor of the need for mechanical ventilation within 14 days of disease onset?
  *Description of the predictor*: Diaphragmatic weakness is a major contributor to respiratory failure in patients with GBS. Neck flexion weakness correlates fairly closely with the degree of diaphragmatic weakness [19]. The utility of bedside neck flexion strength testing as a predictor of respiratory muscle weakness has also been reported in myasthenia gravis [57].
  *Recommendation*: When counseling patients and/or family members/surrogates of patients with acute GBS, we suggest that neck weakness (assessed on presentation or during the early course of hospital admission) alone not be considered a reliable predictor of the need for mechanical ventilation within 14 days of disease onset (weak recommendation; very low-quality evidence).
  *Rationale*: The body of evidence was downgraded for risk of bias, with various studies demonstrating potential bias in the QUIPS domains of study attrition, prognostic factor measurement, outcome measurement, and statistical analysis and reporting, rendering most studies with overall at least moderate risk of bias. The body of evidence was further limited by imprecision due to large confidence intervals, indirectness (due to assessment of neck weakness as part of the overall cranial nerve evaluation in some studies and ability to lift head and neck in others), and inconsistency. Although several studies identified neck weakness as an independent predictor for the need of mechanical ventilation [41, 44, 46, 58], others did not [37, 43, 49], or the data were derived from studies with small sample sizes [59].
5. Question 5: When counseling patients or surrogates of critically ill patients with GBS, should autonomic dysfunction be considered a reliable predictor of the need for mechanical ventilation within 14 days of disease onset?
  *Description of the predictor*: Autonomic dysfunction includes a variety of signs of dysfunction of sympathetic and parasympathetic nervous systems, including tachycardia or bradycardia, hypertension or hypotension, facial flushing, anhidrosis or diaphoresis, urinary retention, or diarrhea or constipation [29, 60]. The definition and assessment of autonomic dysfunction in published studies assessing this predictor mostly centers around cardiovascular autonomic dysfunction [43, 61].
  *Recommendation*: When counseling patients and/or family members/surrogates of patients with acute GBS, we suggest that autonomic dysfunction (assessed during the early course of hospital admission) alone not be considered a reliable predictor of the need for mechanical ventilation within 14 days of disease onset (weak recommendation; very low-quality evidence).
  *Rationale*: The body of evidence was downgraded for risk of bias, with various studies demonstrating potential bias in the QUIPS domains of study attrition, prognostic factor measurement, outcome measurement, and statistical analysis and reporting, rendering many studies with high risk of bias for this factor. The body of evidence was further limited by imprecision with large confidence intervals and inconsistency. Inconsistency among the studies evaluating autonomic dysfunction renders approximately half of the studies finding an association [43, 45, 51, 58, 62], whereas the other half does not [40–42, 49, 50]. In addition to the retrospective nature of many of the data, the primary methodological problem limitation was the absence of a standardized definition of autonomic dysfunction, creating a prominent source of bias in the domain of predictor measurement.

#### Outcome: Independent Ambulation at 3 months or Later

1. Question 1: When counseling patients or surrogates of critically ill patients with GBS, should the disability grade at nadir be considered a reliable predictor of the inability to ambulate independently at 3 months or later?
  *Recommendation*: When counseling family members and/or surrogates of patients with acute GBS, we suggest that the disability grade at nadir during the acute presentation (assessed as worst MRC sum score or GBS disability score within 2 weeks) not be considered a reliable predictor of the inability to ambulate independently at 3 months or later (weak recommendation; low-quality evidence).
  *Rationale*: The body of evidence was downgraded for risk of bias, with various studies demonstrating potential bias in the QUIPS domains of study attrition, prognostic factor measurement, outcome measurement, and statistical analysis and reporting overall, creating a moderate-quality body of evidence. The body of evidence was further limited by imprecision. Additionally, there are limited data available on this factor; although consistent, the thresholds chosen are variable [52, 55, 63] and preclude use as an individual predictor. Of note, overlap exists with assessment of functional outcome in relation to this predictor. Consideration of values and preferences and under consideration of substantial uncertainty, discussion of this predictor as part of counseling may facilitate expectation setting and preparation for prolonged rehabilitation.
2. Question 2: When counseling patients or surrogates of critically ill patients with GBS, should the patient’s age at presentation be considered a reliable predictor of the inability to ambulate independently at 3 months or later?
  *Recommendation*: When counseling family members and/or surrogates of patients with acute GBS, we suggest that the patient’s age at presentation alone not be considered a reliable predictor of the inability to ambulate independently at 3 months or later (weak recommendation; low-quality evidence).
  *Rationale*: The body of evidence was downgraded for risk of bias, with various studies demonstrating potential bias in the QUIPS domains of study attrition, prognostic factor measurement, outcome measurement, and statistical analysis and reporting, rendering the body of evidence of moderate quality. The body of evidence was further limited by imprecision. There was consistency in finding age as an independent predictor of decreased odds for the ability to independently ambulate at 3 months or later [55, 63–65]. Assessment of age groups, however, varied significantly between studies, with some using age group brackets [63], a defined age cutoff [55], or continuous assessment [64, 65] with variable effect size. Hence, recommending a specific age cutoff suitable as an individual predictor for independent ambulation was not deemed feasible.
3. Question 3: When counseling patients or surrogates of critically ill patients with GBS, should the speed of progression of motor weakness during the acute presentation be considered a reliable predictor for independent ambulation at 3 months or later?
  *Recommendation*: When counseling family members and/or surrogates of patients with acute GBS, we suggest that the progression of motor weakness assessed during the acute presentation alone not be considered a reliable predictor of the inability to ambulate independently at 3 months or later (weak recommendation; very low-quality evidence).
  *Rationale*: The body of evidence was downgraded for risk of bias, with various studies demonstrating potential bias in the QUIPS domains of study attrition, prognostic factor measurement, outcome measurement, and statistical analysis and reporting, rendering the body of evidence of low quality. The body of evidence was further limited by imprecision and indirectness. Indirectness was due to the time from symptom onset to presentation or admission, serving as a surrogate for speed of progression in several studies. The body of evidence on this factor is slim, with only two studies reporting a positive association [55, 65]; these two studies used different definitions of speed of progression, i.e., rapid onset of weakness in ≤ 4 days [55] and progression to bedbound state within 2 days [65]. With the further limitations of lack of knowledge on exact disease onset and time from onset to presentation in both of those studies, establishing a specific time frame for the definition of “rapid progression of motor weakness” was not deemed feasible based on the available data.

#### Outcome: Functional Outcome at 6 months or Later

1. Question 1: When counseling patients or surrogates of critically ill patients with GBS, should the need for mechanical ventilation be considered a reliable predictor of poor functional outcome assessed at 6 months or later?
  *Description of the predictor*: The need for mechanical ventilation mostly refers to endotracheal intubation and subsequent mechanical ventilation, without further differentiation between successful extubation versus prolonged mechanical ventilation requiring placement of a tracheostomy tube.
  *Recommendation*: When counseling family members and/or surrogates of patients with acute GBS, we suggest that the need for mechanical ventilation during the acute presentation alone not be considered a reliable predictor of poor functional outcome assessed at 6 months or later (weak recommendation; low-quality evidence).
  *Rationale*: The body of evidence was downgraded for risk of bias, with various studies demonstrating potential bias in the QUIPS domains of study attrition, prognostic factor measurement, outcome measurement, and statistical analysis and reporting, rendering the body of evidence of moderate quality. The body of evidence was further limited by inconsistency, with two studies that examined this association [62, 66] finding that mechanical ventilation was not significantly related to functional outcome, whereas others corroborated a significant association [45, 65, 67, 68]. Additional studies using individual functional outcome definitions also found an association with the requirement for tracheostomy and higher rates of disability [15]. This predictor could not be recommended as moderately reliable because of inconsistency in the body of literature not clearly explained by variation in study characteristics. The need for mechanical ventilation does, however, predict a longer and more complicated inpatient stay and the likelihood of intensive-care-unit-acquired complications [69]. It is reasonable to therefore set expectations regarding the potential duration and complexity of hospitalization and rehabilitation while counseling patients who require mechanical ventilation and their surrogates.
2. Question 2: When counseling patients or surrogates of critically ill patients with GBS, should an axonal electrophysiologic subtype as determined during the acute presentation be considered a reliable predictor of poor functional outcome assessed at 6 months or later?
  *Description of the predictor*: GBS subgroups include acute inflammatory demyelinating polyradiculoneuropathy, acute motor axonal neuropathy, and acute motor and sensory axonal neuropathy, with neurophysiological differentiation of axonal and demyelinating subtypes [12]. Electrophysiologic definitions for axonal patterns have been published and modified over time [12, 70–73]. There is no standardized single definition of axonal subtype, and there is variable use of the aforementioned electrophysiologic criteria. Additionally, there is no standardized requirement for site and number of motor and sensory nerve conduction studies performed per limb. No consistent time point for neurophysiologic assessment has been defined.
  *Recommendation*: When counseling family members and/or surrogates of patients with acute GBS, we suggest that an axonal electrophysiologic subtype during the acute presentation not be considered a reliable predictor of poor functional outcome assessed at 6 months or later (weak recommendation; very low-quality evidence).
  *Rationale*: The body of evidence was downgraded for risk of bias, with various studies demonstrating potential bias in the QUIPS domains of study attrition, prognostic factor measurement, outcome measurement, and statistical analysis and reporting. Imprecision was present, with wide confidence intervals in several studies. This predictor did not meet criteria for reliability or moderate reliability because of inconsistency in the body of evidence, largely manifested in earlier studies. Importantly, a landmark study with electrophysiologic definitions that have since been widely adopted found no relationship between an axonal pattern and unfavorable outcomes [12]. Furthermore, an axonal pattern is more common in Asia [7], where disease outcomes may differ. A study from China found no difference in recovery rates between patients with axonal and those with demyelinating forms of the disease [74]. In a more recent study [75], in additional to an axonal subtype, a demyelinating subtype with low compound muscle action potential (CMAP) amplitude and peroneal nerve studies with low CMAP amplitude were also associated with poor outcomes.
  An important limitation of this predictor is the absence of standardized criteria to identify an axonal electrophysiological pattern in GBS. It should be noted also that there are several possible mechanisms for a reduction in CMAP amplitude, including demyelinating or axonal conduction blocks, primary axonal degeneration, and secondary axonal degeneration [76]. In addition, there is variability in the classification of electrophysiological patterns across studies. Whereas some studies classify electrophysiological patterns as demyelinating or axonal [49], others classify patterns as demyelinating, axonal, or mixed [77]. There is also variation in evaluation of the axonal subtypes, acute motor axonal neuropathy and acute motor and sensory axonal neuropathy [78]. Another factor to consider is that about 10% of patients initially diagnosed with a demyelinating pattern may subsequently demonstrate an axonal pattern [79]. Additional potential confounders include the impact of age on the rate of axonal regeneration (particularly with earlier time points of outcome assessment) and the impact of specific variants, such as acute motor axonal neuropathy, an axonal subtype associated with *Campylobacter jejuni* and diarrhea [52, 70, 80, 81].
  Given these limitations, an axonal electrophysiological subtype was not considered be a reliable or moderately reliable predictor despite a majority of studies demonstrating an independent association with unfavorable outcome [49, 52, 75, 77, 78, 82].
3. Question 3: When counseling patients or surrogates of critically ill patients with GBS, should the patient’s age at the time of hospital admission be considered a reliable predictor of poor functional outcome assessed at 6 months or later?
  *Recommendation*: When counseling family members and/or surrogates of patients with acute GBS, we suggest that the patient’s age alone not be considered a reliable predictor of poor functional outcome assessed at 6 months or later (weak recommendation; low-quality evidence).
  *Rationale*: The body of evidence was downgraded for risk of bias, with various studies demonstrating potential bias in the QUIPS domains of study attrition, prognostic factor measurement, outcome measurement, and statistical analysis and reporting, rendering many studies with overall at least moderate risk of bias. The body of evidence was further limited by imprecision. The studies evaluating age as a prognostic factor for outcome [20, 77, 82, 83] either used age as a continuous variable [15, 20] or used dichotomized age groups varying between 40 [66, 83] and 70 years [82]. Of note, one study (with 32 study participants) found that once older patients survived the early, most critical period, recovery was often as good as for younger patients [84]. As such, there are not sufficient data to indicate a specific age cutoff to consider when prognosticating. Importantly, the effect size for age as a predictor also varied considerably between studies. Hence, age should not be used in isolation as a predictor.
4. Question 4: When counseling patients or surrogates of critically ill patients with GBS, should the patient’s disability grade at disease nadir be considered a reliable predictor of poor functional outcome?
  *Description of the predictor*: The disability grade at nadir is assessed variably, largely with the GBSDS or through the MRC sum score.
  *Recommendation*: When counseling family members and/or surrogates of patients with acute GBS, we suggest that the patient’s disability grade at disease nadir not be considered a reliable predictor of poor functional outcome assessed at 6 months or later (weak recommendation; low-quality evidence).
  *Rationale*: The body of evidence was downgraded for risk of bias, with various studies demonstrating potential bias in the QUIPS domains of study attrition, prognostic factor measurement, outcome measurement, and statistical analysis and reporting, rendering many studies with high risk of bias for this factor. The body of evidence was further limited by imprecision, with large confidence intervals, and indirectness based on the assessments for disability, which included crude assessments such as “bed bound” or “nonambulatory” as opposed to using a defined disability measure. Two studies assessed the level of disability by the MRC sum score [49, 85], whereas the remainder used the GBSDS either continuously or dichotomized [22, 66, 77, 78, 86, 87]. Effect size varied considerably, and confidence intervals were large in some analyses [78, 85].

### Recommendations: clinical prediction models

#### Outcome: mechanical ventilation within 14 days of disease onset

1. Question 1: When counseling patients or surrogates of critically ill patients with GBS, should the EGRIS be considered a reliable predictor of the need for mechanical ventilation within 14 days of disease onset?
  *Description of the predictor*: The EGRIS estimates the risk of respiratory failure, defined by the need for mechanical ventilation within the first week from hospital admission. The score was developed in 397 patients from a randomized trial and validated in a Dutch GBS cohort study [38]. It was further validated in three smaller cohorts [88–90] as well as in the large International GBS Outcome Study (IGOS) of 1023 patients [91]. Its prediction is based on three clinical variables determined at hospital admission (see Table 4). The sum score ranges from 0 to 7, corresponding to a predicted risk of respiratory failure from 1 to 90%. The EGRIS is also available as an online tool that can be accessed at https://gbstools.erasmusmc.nl/prognosis-tool/0/0.
  *Recommendation*: When counseling family members and/or surrogates of patients with acute GBS, we suggest that the EGRIS be considered a moderately reliable prognostic model for the probability of needing mechanical ventilation within 14 days of disease onset (weak recommendation; moderate-quality evidence).
  *Rationale*: The body of evidence was downgraded for risk of bias, with various studies demonstrating potential bias in the PROBAST domains of participant selection, study attrition, prognostic factor measurement, and calibration. In the IGOS cohort, AUC values were > 80% for all validation subgroups, but observed proportions of mechanical ventilation were lower (10%) than predicted risks (21%), requiring recalibration [91]. Of note, in a Peruvian cohort study, the EGRIS showed only moderate discrimination capacity, with an AUC of 0.63 [90]. In addition, only prediction of mechanical ventilation within 1 week from hospital admission is available. However, only 3% of patients in the development cohort were intubated after the first week of admission.
2. Question 2: When counseling patients or surrogates of critically ill patients with GBS, should the Sharshar model be considered a reliable predictor of the need for mechanical ventilation within 14 days of disease onset?
  *Description of the predictor*: The Sharshar model was developed in 722 patients enrolled in two randomized clinical trials by the French Cooperative Group on Plasma Exchange in GBS [44] and validated in 92 patients with GBS admitted consecutively to the neurology service of a tertiary care teaching hospital in India [92]. Sharshar et al. identified six clinical variables, including admission within 7 days of onset, inability to lift head and elbows, inability to stand, ineffective cough, and elevated liver enzyme levels. Each variable was given 1 point if present, and the sum score ranges from 0 to 6. Mechanical ventilation was required in > 85% of patients with at least four predictors [44].
  *Recommendation*: When counseling family members and/or surrogates of patients with acute GBS, we suggest that the Sharshar model not be considered a reliable prognostic model for the probability of needing mechanical ventilation within 14 days of disease onset (weak recommendation; low-quality evidence).
  *Rationale*: The body of evidence was downgraded for risk of bias, with the development and validation studies demonstrating potential bias in the PROBAST domains of participant selection, study attrition, prognostic factor measurement, outcome measurement, and statistical analysis and reporting, rendering the body of evidence of low quality. The body of evidence was further limited by imprecision. The body of evidence on this prognostic model is also insufficient, with only one small validation study reporting calibration results [92].
3. Question 3: When counseling patients or surrogates of critically ill patients with GBS, should the Ning Score (MVN) be considered a reliable predictor of the need for mechanical ventilation within 14 days of disease onset?
  *Description of the predictor*: The Ning Score (MVN) was developed and validated In two Chinese cohorts of 312 and 114 patients with GBS and consists of the following variables: hospital stay > 14 days, glossopharyngeal and vagal nerve deficits, Hughes functional grading scale scores at admission, and the neutrophil/lymphocyte ratio [93].
  *Recommendation*: When counseling family members and/or surrogates of patients with acute GBS, we suggest that the Ning Score (MVN) not be considered a reliable prognostic model for the probability of needing mechanical ventilation within 14 days of disease onset (weak recommendation; low-quality evidence).
  *Rationale*: The body of evidence was downgraded for risk of bias, with the development and validation studies demonstrating potential bias in the PROBAST domains of study attrition, prognostic factor measurement, outcome measurement, and statistical analysis and reporting, rendering the body of evidence of low quality. The body of evidence on this prognostic model is insufficient, with only one validation study reporting calibration results [93].

**Table 4:** Erasmus Guillain–Barré Syndrome Respiratory Insufficiency Score

Predictor	Categories	Score
Time from onset of weakness to hospital admission, days	> 7	0
	4–7	1
	≤ 3	2
Facial and/or bulbar weakness at hospital admission	Absent	0
	Present	1
Medical Research Council sum score at hospital admission	51–60	0
	41–50	1
	31–40	2
	21–30	3
	≤ 20	4
Total score		0–7

#### Outcome: Independent Ambulation Assessed at ≥ 3 months

1. Question 1: When counseling patients or surrogates of critically ill patients with GBS, should the EGOS within 2 weeks of admission be considered a reliable predictor of the need for independent ambulation at 3 months or later?
  *Description of the predictor*: The EGOS was developed and validated in 374 patients from two randomized controlled trials as well as one pilot study and validated in a set of 379 patients from another randomized trial [63] as well as one smaller single-center cohort. The score predicts the risk of being unable to walk independently at 6 months of GBS onset based on the age, preceding diarrhea, and GBS disability score at 2 weeks after entry, with scores ranging from 1 to 7 (see Table 5).
  *Recommendation*: When counseling family members and/or surrogates of patients with acute GBS, we suggest that the EGOS within 2 weeks of admission be considered a moderately reliable predictor for the probability of independent ambulation by 3 months or later (weak recommendation; moderate-quality evidence).
  *Rationale*: The body of evidence was downgraded for risk of bias, with various studies demonstrating potential bias in the PROBAST domains of participant selection, study attrition, prognostic factor measurement, and calibration. In addition, only outcome prediction at 6 months is available.
2. Question 2: When counseling patients or surrogates of critically ill patients with GBS, should the mEGOS assessed on admission and during the early course of hospital admission be considered a reliable predictor of independent ambulation by 3 months or later?
  *Description of the predictor*: The mEGOS was developed and validated from a Dutch GBS cohort study [94] and further validated in two smaller cohorts [88, 89] as well the large IGOS consisting of 809 patients [32]. The score predicts the risk of being unable to walk independently at 4 weeks, 3 months, and 6 months of GBS onset based on the variables age, muscle strength, and preceding diarrhea and can be assessed either at hospital admission or at day 7 of admission (see Table 6). The mEGOS is also available as an online tool that can be accessed at https://gbstools.erasmusmc.nl/prognosis-tool/0/0.
  *Recommendation*: When counseling family members and/or surrogates of patients with acute GBS, we suggest that the mEGOS at hospital admission and at 1 week be considered a moderately reliable predictor for the probability of independent ambulation by 3 months or later (weak recommendation; moderate-quality evidence).
  *Rationale*: The body of evidence was downgraded for risk of bias, with various studies demonstrating potential bias in the PROBAST domains of participant selection, study attrition, prognostic factor measurement, and calibration. In the IGOS cohort, AUC values were > 70% for all validation subgroups, but observed outcomes were either worse (in Europe/North America) or better (Asia) than predicted. Recalibration improved model accuracy and led to a region-specific version. Furthermore, predictive accuracy is lower if the mEGOS is assessed on hospital admission compared with at day 7 of admission [32].

**Table 5:** Erasmus Guillain–Barré Syndrome Outcome Score

Predictor	Categories	Score
Age at onset, years	> 60	1
	41–60	0.5
	≤ 40	0
Diarrhea (≤ 4 weeks)	Absent	0
	Present	1
Guillain–Barré syndrome disability score (at 2 weeks after entry)	0 or 1	1
	2	2
	3	3
	4	4
	5	5
Total score		1–7

**Table 6:** Modified Erasmus Guillain–Barré Syndrome Outcome Score (mEGOS)

mEGOS at hospital admission		mEGOS at day 7 of admission	
Prognostic factors	Score	Prognostic factors	Score
Age at onset, years		Age at onset, years	
≤ 40	0	≤ 40	0
41–60	1	41–60	1
> 60	2	> 60	2
Preceding diarrhea		Preceding diarrhea	
Absent	0	Absent	0
Present	1	Present	1
MRC sum score at hospital admission		MRC sum score at day 7 of admission	
51–60	0	51–60	0
41–50	2	41–50	3
31–40	4	31–40	6
≤ 30	6	≤ 30	9
Total score	0–9	Total score	0–12

*MRC* Medical research council

#### Outcome: Functional Outcome Assessed at ≥ 6 months

1. Question 1: When counseling patients or surrogates of critically ill patients with GBS, should the EGOS as assessed during the acute course of hospitalization be considered a reliable predictor of functional outcome at 6 months or later?
  *Recommendation*: When counseling family members and/or surrogates of patients with acute GBS, we suggest that the EGOS not be considered a reliable predictor of functional outcome (weak recommendation; low-quality evidence).
  *Rationale*: The EGOS was not developed for prediction of functional outcome, and only two smaller studies have assessed the predictive value of the EGOS for functional outcome with low accuracy [88, 89].
2. Question 2: When counseling patients or surrogates of critically ill patients with GBS, should the mEGOS as assessed during the acute course of hospitalization be considered a reliable predictor of functional outcome assessed at 6 months or later?
  *Recommendation*: When counseling family members and/or surrogates of patients with acute GBS, we suggest that the mEGOS not be considered a reliable predictor of functional outcome (weak recommendation; low-quality evidence).
  *Rationale*: The mEGOS was not developed for prediction of functional outcome, and only one small study has assessed the predictive value of the mEGOS for functional outcome with low accuracy [89].
3. Question 3: When counseling patients or surrogates of critically ill patients with GBS, should the EGRIS as assessed during the acute course of hospitalization be considered a reliable predictor of functional outcome assessed at 6 months or later?
  *Recommendation*: When counseling family members and/or surrogates of patients with acute GBS, we suggest that the EGRIS not be considered a reliable predictor of functional outcome (weak recommendation; low-quality evidence).
  *Rationale*: The EGRIS was not developed for prediction of functional outcome, and only two smaller studies have assessed the predictive value of the EGRIS for functional outcome with low accuracy [88, 89].

#### Future Directions

**Fig. 2:**
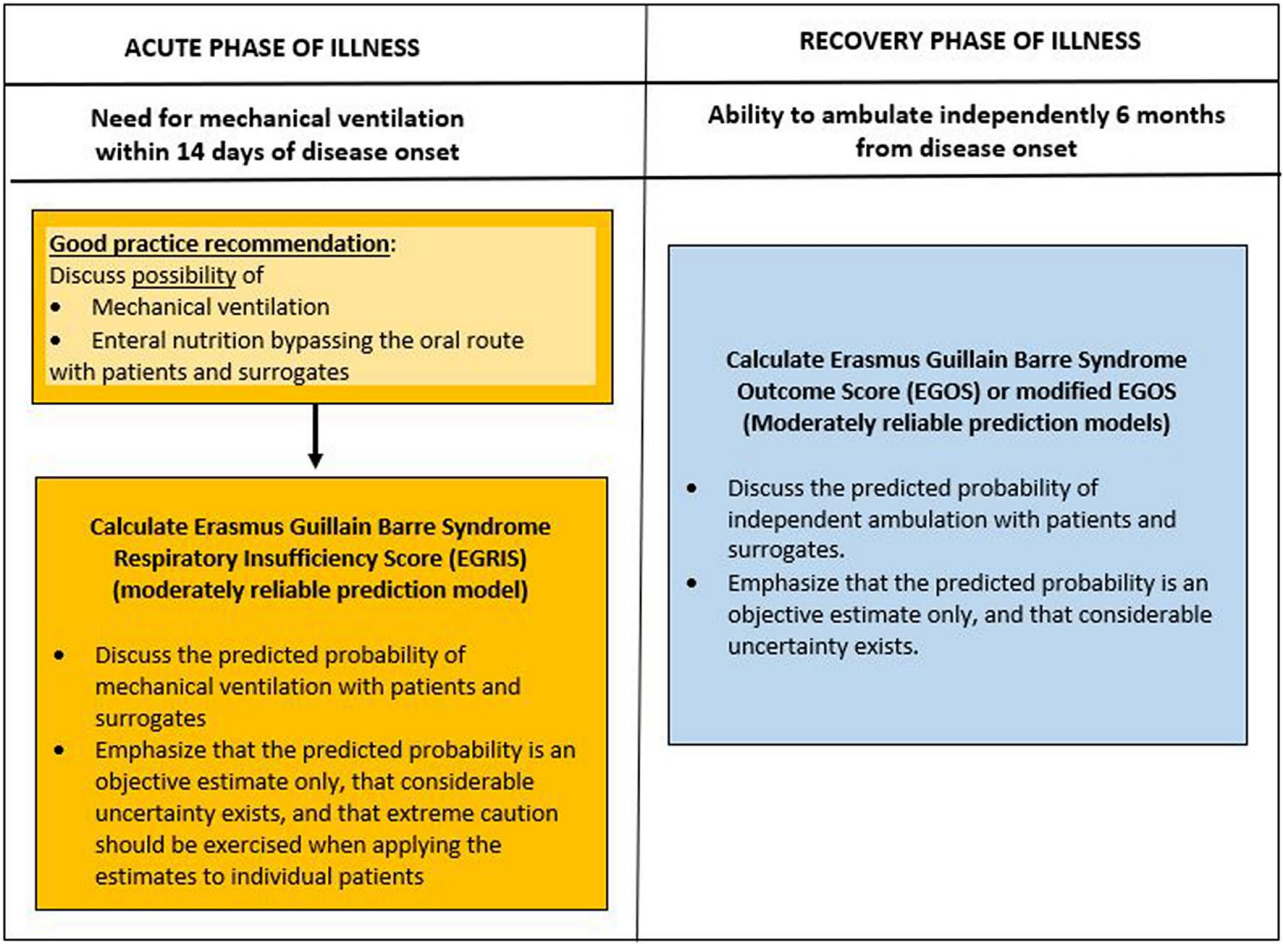
Neuroprognostication in Guillain–Barré syndrome

Only a limited number of predictors had a sufficient body of evidence to support recommendations for use in clinical practice. A suggested approach to neuroprognostication in GBS is shown in Fig. 2. Although these predictors met criteria for use in prognostication, limitations are considerable, including lack of standardized definitions, lack of standardized assessment methods, and variable effect size. Therefore, many patients will have an indeterminate prognosis on the basis of these guidelines, highlighting the importance of future high-quality neuroprognostication research. Based on the most common study limitations identified in our systematic review, future studies should consider the following general principles:

1. Outcomes and predictors should be assessed using standardized definitions with data sets for the acute, subacute, and chronic phases of the disease. Definitions subject to the least interrater variance should be given preference. For example, criteria for intubation should ideally be standardized when the need for mechanical ventilation is studied either as an outcome or as a predictor. Similarly, standardized electrophysiologic criteria should be used.
2. Outcomes should be assessed at hospital discharge, at 3, 6, and 12 months and beyond to include long-term outcomes and assess trends over time.
3. Assessors of long-term outcomes should ideally be blinded to the initial severity and clinical course of the patient.
4. Patients who did not receive standard-of-care immunomodulatory therapy should be excluded from prognostication studies.
5. Future studies should be based on larger sample sizes that stem from multiple centers and should ideally be conducted internationally.

Discussions with patient and family representatives highlighted the importance of patient-centered outcomes following GBS that have not been studied rigorously yet. This includes pain [35], social interactions, and employment [95]. Patients and surrogates should also receive counseling and advice related to navigation of the health care system, particularly the transition from the acute phase to subacute and chronic phases, as well as health care costs [28, 96]. Because intensive rehabilitation may improve outcome [25, 26], future studies should further examine the impact of rehabilitation on outcomes [26, 97]. Similarly, factors such as nutrition may play a role that is yet to be elucidated [27]. Biomarker research has largely focused on electrophysiological studies but may include different biomarkers in the future [98]. For both traditionally examined and additional domains, future prospective studies should use standardized instruments and time points for evaluation and compare occurrence with that of an age- and sex-matched control population.

## Conclusions

These guidelines provide recommendations on the use of predictors of clinical outcomes in GBS in the context of counseling patients and surrogates and suggest broad principles of neuroprognostication. Few predictors were considered moderately reliable based on the available body of evidence, and higher quality data are needed.

### Endorsements

These guidelines were endorsed by the Society of Critical Care Medicine. The American Academy of Neurology affirms the value of these guidelines.

### Supplementary Information

Below is the link to the electronic supplementary material.

Supplementary file1 (DOCX 38 KB)

Supplementary file2 (DOCX 19 KB)

Supplementary file3 (XLSX 40 KB)
