# Author Correction: Guidelines for Neuroprognostication in Adults with Guillain–Barré Syndrome

**DOI:** 10.1007/s12028-023-01830-1

**Published:** 2023-09-19

**Authors:** Katharina M. Busl, Herbert Fried, Susanne Muehlschlegel, Katja E. Wartenberg, Venkatakrishna Rajajee, Sheila A. Alexander, Claire J. Creutzfeldt, Gabriel V. Fontaine, Sara E. Hocker, David Y. Hwang, Keri S. Kim, Dominik Madzar, Dea Mahanes, Shraddha Mainali, Juergen Meixensberger, Oliver W. Sakowitz, Panayiotis N. Varelas, Thomas Westermaier, Christian Weimar

**Affiliations:** 1 Departments of Neurology and Neurosurgery, College of Medicine, University of Florida, Gainesville, FL USA; 2 Department of Neurosurgery, Denver Health Medical Center, Denver, CO USA; 3 Departments of Neurology, Anesthesiology, and Surgery, University of Massachusetts Chan Medical School, Worcester, MA USA; 4 Department of Neurology, University of Leipzig, Leipzig, Germany; 5 Departments of Neurology and Neurosurgery, University of Michigan, Ann Arbor, MI USA; 6 School of Nursing, University of Pittsburgh, Pittsburgh, PA USA; 7 Department of Neurology, University of Washington, Seattle, WA USA; 8 Departments of Pharmacy and Neurosciences, Intermountain Health, Salt Lake City, UT USA; 9 Department of Neurology, Mayo Clinic, Rochester, MN USA; 10 Department of Neurology, University of North Carolina, Chapel Hill, NC USA; 11 Department of Pharmacy Practice, University of Illinois, Chicago, IL USA; 12 Department of Neurology, University of Erlangen-Nuremberg, Erlangen, Germany; 13 Departments of Neurology and Neurosurgery, University of Virginia Health, Charlottesville, VA USA; 14 Department of Neurology, Virginia Commonwealth University, Richmond, VA USA; 15 Department of Neurosurgery, University of Leipzig, Leipzig, Germany; 16 Department of Neurosurgery, Neurosurgery Center Ludwigsburg-Heilbronn, Ludwigsburg, Germany; 17 Department of Neurology, Albany Medical College, Albany, NY USA; 18 Department of Neurosurgery, University of Würzburg, Würzburg, Germany; 19 Institute of Medical Informatics, Biometry, and Epidemiology, University Hospital Essen, Essen and BDH-Clinic Elzach, Essen, Germany; 20 BDH-Clinic Elzach, Elzach, Germany

**Author Correction: Neurocritical Care (2023) 38:564–583** 10.1007/s12028-023-01707-3

The original article has been updated to insert the following sentence, before the line “The librarian search string..” in the Systematic Review Methodology paragraph.

The in-depth literature search first conducted on February 20, 2019, included literature from 1946 onwards and was last updated on 3/16/2022.

