# Supplementary material for: Guidelines for Neuroprognostication in Adults with Guillain–Barré Syndrome
Source: Neurocrit Care. 2023 Mar 25;38(3):564–83. doi: 10.1007/s12028-023-01707-3 (PMC10241707; doi:10.1007/s12028-023-01707-3)
Supplement: Supplementary file 2 — Supplementary file2 (DOCX 19 KB) [file 12028_2023_1707_MOESM2_ESM.docx]

**SUPPLEMENTARY APPENDIX 4**

**Librarian search string – Guillain-Barré-Syndrome**

Database: All Ovid Medline <1946 - present>

Search Strategy:

--------------------------------------------------------------------------------

1 exp Guillain-Barre Syndrome/ (4664)

2 (Guillain-Barre adj3 Syndrome*).tw. (8306)

3 (Guillain Barre adj3 Syndrome*).tw. (8306)

4 Polyradiculoneuropath*.tw. (1460)

5 acute autoimmune neuropath*.tw. (6)

6 acute inflammatory polyneuropath*.tw. (47)

7 acute inflammatory demyelinating polyneuropath*.tw. (294)

8 AIDP.tw. (292)

9 acute motor axonal neuropath*.tw. (326)

10 (polyneuropathies adj3 (acute or inflammatory)).tw. (170)

11 AMAN.tw. (343)

12 (miller adj3 fisher).ti,ab. (781)

13 or/1-12 (11035)

14 exp Analysis of Variance/ [includes Multivariate Analysis] (326771)

15 multivariate.tw. (293720)

16 treatment outcome/ (885811)

17 outcome.tw. (887975)

18 mortality/ or mo.fs. (563611)

19 "Predictive Value of Tests"/ (188131)

20 cognition.mp. [mp=title, abstract, original title, name of substance word, subject heading

word, floating sub-heading word, keyword heading word, organism supplementary concept word,

protocol supplementary concept word, rare disease supplementary concept word, unique

identifier, synonyms] (182988)

21 Disability Evaluation/ (44760)

22 mortality/ or mo.fs. (563611)

23 (predict* or predictor* or prediction*).tw. (1405701)

24 prognostic*.tw. (268771)

25 prognos*.tw. (536821)

26 exp Quality of Life/ (171899)

27 "Quality of life".tw. (241815)

28 Recovery of Function/ (46471)

29 scale.ti,ab. (629667)

30 score.ti,ab. (478944)

31 scoring tool*.tw. (605)

32 Survival/ (4559)

33 Time factors/ (1143362)

34 14 or 16 or 17 or 18 or 19 or 23 or 24 or 25 or 26 or 29 or 30 or 31 or 32 or 33 (5199035)

35 13 and 34 (2282)

36 prognosis.sh. or diagnosed.tw. or cohort:.mp. or predictor:.tw. or death.tw. or exp models,

statistical/ [validated hedge from the Health Information Research Unit, McMaster University]

(2446889)

37 13 and 36 (1542)

38 35 or 37 (3116)

39 exp cohort studies/ [includes: follow-up studies/, longitudinal studies/, prospective studies/,

retrospective studies/, controlled before-after studies/, cross-sectional studies/, or historically

controlled study/] (1824723)

40 (Follow-up or longitudinal or prospective or retrospective or before-after or cross-sectional

or controlled).tw. (2597128)

41 exp registries/ (84199)

42 (predict* or predictor or prognos* or prognost*).ti. (415631)

43 or/39-42 (3683729)

44 13 and 38 and 43 (1157)

45 animals/ not (humans/ and animals/) (4515460)

46 limit 44 to ("newborn infant (birth to 1 month)" or "infant (1 to 23 months)" or "preschool child

(2 to 5 years)" or "child (6 to 12 years)") (309)

47 *pediatrics/ (36063)

48 case reports.pt. (1923094)

49 letter.pt. (1015878)

50 45 or 46 or 47 or 48 or 49 (7221430)

51 44 not 50 (740)

***************************
